# Characterization of Musclin as a New Target for Treatment of Hypertension

**DOI:** 10.1155/2014/354348

**Published:** 2014-03-09

**Authors:** Jia-Wei Lin, Cheng-Chia Tsai, Li-Jen Chen, Ho-Shan Niu, Chen Kuei Chang, Chiang-Shan Niu

**Affiliations:** ^1^Department of Neurosurgery, College of Medicine, Taipei Medical University and Shuang Ho Hospital, Taipei Medical University, Taipei City 10361, Taiwan; ^2^Graduate Institute of Disease Prevention and Control, College of Medicine, Taipei Medical University and Shuang Ho Hospital, Taipei Medical University, Taipei City 10361, Taiwan; ^3^Institute of Basic Medical Sciences, College of Medicine, National Cheng Kung University, Tainan City 70101, Taiwan; ^4^Department of Nursing, Tzu Chi College of Technology, Hualien City 97005, Taiwan

## Abstract

Musclin is a novel skeletal muscle-derived factor found in the signal sequence trap of mouse skeletal muscle cDNAs. Recently, it has been demonstrated that musclin is involved in the pathogenesis of spontaneously hypertensive rats (SHRs). However, it is known as a genetic hypertension model. In the present study, we aim to investigate the role of musclin in another animal model of hypertension and characterize the direct effect of musclin on vascular contraction. The results show that expression of musclin was increased in arterial tissues isolated from DOCA-salt induced hypertensive rats or the normal rats received repeated vasoconstriction with phenylephrine. Additionally, direct incubation with phenylephrine did not modify the expression of musclin in the *in vitro* studies. Also, the direct effect of musclin on the increase of intracellular calcium was observed in a concentration-dependent manner. These results provide the evidence to support that musclin is involved in hypertension. Thus, musclin is suitable to be considered as a novel target for treatment of hypertension.

## 1. Introduction

Hypertension is a cardiovascular risk factor and a major healthcare problem [1]. So far, although it is well known that the vasculature, kidney, skeletal muscle, and central nervous system contribute to the development of hypertension, the mechanisms for the progression of higher blood pressure are still not completely clarified [1]. Basically, both human hypertension and experimental models of hypertension are mainly characterized by increased intravascular pressure that causes constriction of vascular smooth muscle cells (VSMCs) in resistant arteries, and this response, known as myogenic tone, is a key element for the maintenance of blood pressure [2, 3]. Moreover, this myogenic response, which has also been demonstrated to occur independently of neural control in isolated vessels, is considered to be an intrinsic function of the smooth muscle vessel wall [4].

Musclin is a novel muscle-derived secretory peptide found in the signal sequence trap of mouse skeletal muscle cDNAs. Musclin mRNA was almost exclusively expressed in the skeletal muscle of rodents and obesity models [5]. The function of musclin has been described as responsive to insulin* in vivo* and inducing insulin resistance* in vitro* [6, 7]. Furthermore, musclin is also known as a bone-active molecule that is highly expressed in cells of the osteoblast lineage of animals [5, 8].

Recently, a higher expression of musclin in arterial tissue has been observed in spontaneous hypertensive rats (SHRs) [9]. Then, authors claimed that musclin is involved in the pathogenesis of hypertension. However, SHR is known as a genetic disorder of hypertension. Experiments by using of different hypertensive animal model will be helpful to identify the role of musclin in the development of hypertension. The main aim of this study is to investigate the expression of musclin in other hypertensive animal models and characterize the potential mechanism(s) for musclin induced hypertension.

## 2. Material and Methods

### 2.1. Animals

Eight-week-old male Wistar rats, weighing from 250 to 280 g, were obtained from the Animal Center of National Cheng Kung University Medical College. The rats were housed individually in plastic cages under standard laboratory conditions. They were kept under a 12 h light/dark cycle and had free access to food and water. All experiments were performed under anesthesia with 2% isoflurane, and all efforts were made to minimize the animals' suffering. The animal experiments were approved and conducted in accordance with local institutional guidelines for the Care and Use of Laboratory Animals in Chi-Mei Medical Center, and the experiments conformed to the Guide for the Care and Use of Laboratory Animals as well as the guidelines of the Animal Welfare Act.

### 2.2. Deoxycorticosterone Acetate and Sodium Chloride (DOCA-Salt) Induced Hypertensive Rats

According to previous reports [10–12], Wistar rats were anesthetized and underwent uninephrectomy (small flank incision, right side). One week after surgery, all rats started receiving the subcutaneous injections of DOCA (Sigma-Aldrich, Germany) (20 mg/kg during the first week, 12 mg/kg during the second and third weeks, and 6 mg/kg to the end of treatment) and the drinking water contained 1.0% NaCl and 0.2% KCl. The control rats (vehicle sham) received vehicle injections (1 : 1 mineral oil and propylene glycol) and normal tap water. Each rat was placed into a holder to determine the mean blood pressure (MBP) through a noninvasive tail-cuff monitor (MK2000; Muromachi Kikai, Tokyo, Japan) under conscious and values for each animal were estimated in triplicate. All rats were then sacrificed to isolate the aorta for assay of musclin expression through Western blotting analysis.

### 2.3. Phenylephrine (PE) Induced Hypertension

For challenge with hypertension, Wistar rats were injected intravenously (IV) with phenylephrine (10 *μ*g/kg; Sigma Chemical) dissolved in 9% saline, 4 times daily, for 7 days as described previously [13, 14]. The age-matched rats were divided into three groups (*n* = 8): normal rats (Con), vehicle-treated normal rats (Veh), and PE induced hypertensive rats (PE). After a 7-day treatment, each rat was placed into a holder to determine the mean blood pressure (MBP) through a noninvasive tail-cuff monitor (MK2000; Muromachi Kikai, Tokyo, Japan) under conscious and values for each animal were estimated in triplicate. All rats were then sacrificed to isolate the aorta for assay of musclin expression using Western blotting analysis.

### 2.4. Cell Line and Culture Conditions

Rat cell line for vascular smooth muscle cells (A7r5 cells) (BCRC, Hsinchu, Taiwan) were cultured in RPMI-1640 medium (Gibco BRL, Paisley, Scotland) supplemented with 10% fetal calf serum (FCS) (Biologic Industries, Kibbutz Beit Haemek, Israel), penicillin (100 IU/mL), streptomycin (100 mg/mL) (Sigma, St. Louis, MO, USA), and amphotericin B (2.5 mg/mL, Gibco). The cells were trypsinized (trypsin used was purchased from Gibco) and subcultured once a week, and the medium was changed every 3-4 days. For the experiments, the cells were seeded on round (10 cm diameter) plastic dishes and cultured with PE under the doses of 0.1 *μ*M and 1 *μ*M for 24 hours. Samples were collected for detection of the expression of musclin by Western blotting analysis.

### 2.5. Measurement of Intracellular Calcium Concentrations

Musclin was purchased from Phoenix Pharmaceuticals Inc. (Burlingame, CA, USA). Changes in the intracellular calcium concentration were detected using the fluorescent probe fura-2 [15]. A7r5 cells were placed in buffered physiological saline solution (PSS) containing 140 mM NaCl, 5.9 mM KCl, 1.2 mM CaCl_2_, 1.4 mM MgCl_2_, 11.5 mM glucose, 1.8 mM Na_2_HPO_4_, and 10 mM Hepes-Tris, next, 5 *μ*M fura-2 was added to this solution, and then, the cells were incubated for 1 h in humidified atmosphere containing 5% CO_2_ and 95% air at 37°C. The cells were washed and incubated for further 30 min in PSS. The A7r5 cells were then inserted into a thermostatic (37°C) cuvette containing 2 mL of PSS and various doses of musclin or inhibitor as indicated. The fluorescence was continuously recorded using a fluorescence spectrofluorimeter (Hitachi F-2000, Tokyo, Japan). The values of intracellular calcium ([Ca^2+^]^i^) were calculated from the ratio *R* = *F*340/*F*380 by the formula [Ca^2+^]^i^ = *K*_*d*_ *B* (*R* − *R*_min⁡_)/(*R*_max⁡_−*R*), where *K*_*d*_ is 225 nM, *F* is the fluorescence measured at 340 nm and 380 nm, and *B* is the ratio of fluorescence of the free dye to that of the Ca^2+^-bound dye measured at 380 nm. *R*_max⁡_ and *R*_min⁡_ were determined in separate experiments by using musclin to equilibrate [Ca^2+^]^i^ with ambient [Ca^2+^] (*R*_max⁡_) and adding 0.1 mM MnCl_2_ and 1 mM EGTA (*R*_min⁡_). Background autofluorescence was measured in unloaded cells and was subtracted from all measurements.

### 2.6. Western Blotting Analysis

Protein was extracted from tissue homogenates and cell lysates using ice-cold radioimmunoprecipitation assay (RIPA) buffer supplemented with phosphatase and protease inhibitors (50 mmol/L sodium vanadate, 0.5 mM phenylmethylsulphonyl fluoride, 2 mg/mL aprotinin, and 0.5 mg/mL leupeptin). Protein concentrations were determined with a Bio-Rad protein assay (Bio-Rad Laboratories, Inc., Hercules, CA, USA). Total proteins (30 *μ*g) were separated by SDS/polyacrylamide gel electrophoresis (10% acrylamide gel) using a Bio-Rad Mini-Protein II system. The protein was transferred to the expanded polyvinylidene difluoride membranes (Pierce, Rockford, IL, USA) with a Bio-Rad Trans-Blot system. After transfer, the membranes were washed with PBS and blocked for 1 h at room temperature with 5% (w/v) skimmed milk powder in PBS. The manufacturer's instructions were followed for the primary antibody reactions. Blots were incubated overnight at 4°C with an immunoglobulin-G polyclonal rabbit anti-mouse antibody (Affinity BioReagents, Inc., Golden, CO, USA) (1 : 500) in 5% (w/v) skimmed milk powder dissolved in PBS/Tween 20 (0.5% by volume) to bind the target protein such as musclin. The blots were incubated with goat polyclonal antibody (1 : 1000) to bind the actin which served as the internal control. After the removal of the primary antibody, the blots were extensively washed with PBS/Tween 20 and then incubated for 2 h at room temperature with the appropriate peroxidase-conjugated secondary antibody diluted in 5% (w/v) skimmed milk powder and dissolved in PBS/Tween 20. The blots were developed by autoradiography using an ECL-Western blotting system (Amersham International, Buckinghamshire, UK). The immunoblots of musclin (11 kDa) were quantified with a laser densitometer.

### 2.7. Preparation of Isolated Arterial Strips

The isolated arterial strips from Wistar rats were used. Each rat was sacrificed by decapitation under anesthesia. After the arterial strips had been carefully freed from fat and connective tissue, the spirally cut strips were then mounted in organ baths filled with 10 mL oxygenated Krebs' buffer (95% O_2_, 5% CO_2_) at 37°C containing (in mmol/L) NaCl 135; KCl 5; CaCl_2_ 2.5; MgSO_4_ 1.3; KH_2_PO_4_ 1.2; NaHCO_3_ 20; and d-glucose 10 (pH 7.4). The calcium-free buffer was prepared in the same manner while CaCl_2_ was not included. To exclude a possible role of the endothelium in musclin induced vasoconstriction, the tests were conducted in endothelium-denuded preparations. The endothelium was removed by gently rubbing it against the teeth of a pair of forceps. Successful removal of the endothelium was confirmed by histological identification and failure of 1 *μ*mol/L acetylcholine to relax the rings that had been precontracted with potassium chloride as described previously [16].

Each preparation was connected to strain gauges (FT03; Grass Instrument, Quincy, MA, USA). Isometric tension was recorded using chart software (MLS023, Powerlab; ADInstruments, Bella Vista, NSW, Australia). Strips were mounted and allowed to stabilize for 2 h. Each preparation was then gradually stretched to achieve an optimal resting tension of 1 g. After the tension had stabilized, the arterial strips were exposed to musclin at various concentrations (0.01–10 nmol/L), with a wait time of 15–20 min between all musclin doses. Then, the increase in tonic contraction (vasoconstriction) was evaluated. Once the sample stabilized, oxygenated Krebs buffer was replaced, and then potassium chloride (50 mmol/L) (Sigma-Aldrich, St. Louis, MO, USA) was added as a positive control.

### 2.8. Statistical Analysis

Results were expressed as mean ± SE of each group. Statistical analysis was carried out using ANOVA analysis and Newman-Keuls post hoc analysis. Statistical significance was set as *P* < 0.05.

## 3. Results

### 3.1. Increase of Musclin Expression in Deoxycorticosterone Acetate and Sodium Chloride (DOCA-Salt) Induced Hypertensive Rats

**Figure 1:**
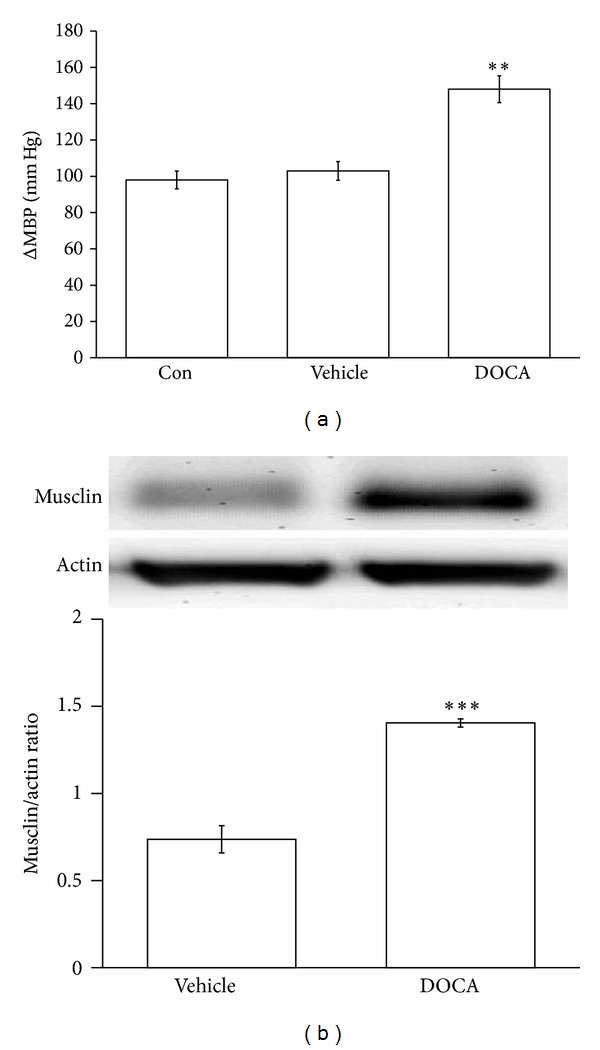
Effect of DOCA-salt induced hypertension on the mean blood pressure and the expression of musclin protein. Wistar rats underwent uninephrectomy and received subcutaneous injections of DOCA salt and drinking water supplemented with 1.0% NaCl and 0.2% KCl (DOCA group). The vehicle-sham rats (vehicle) received vehicle injections (1 : 1 mineral oil and propylene glycol) and normal tap water. The mean blood pressure (MBP) was recorded using a noninvasive tail-cuff monitor (a) while the expression of musclin protein (11 kDa) was determined using Western blotting analysis (b). The quantification of the results is indicated as the means with the SE (*n* = 8 per group) in each column shown in the lower panel. **P < 0.01 and ***P < 0.001 compared with vehicle-sham group.

We examined the expression of musclin in the aorta of DOCA-salt induced hypertensive rats. The mean blood pressure (MBP) in these animals was significantly elevated (Figure 1(a)) as compared to the vehicle-sham group. Also, the expression of musclin in aorta was markedly raised in DOCA-salt induced hypertensive rats (Figure 1(b)).

### 3.2. Increase of Musclin Expression by Phenylephrine Induced Hypertension

**Figure 2:**
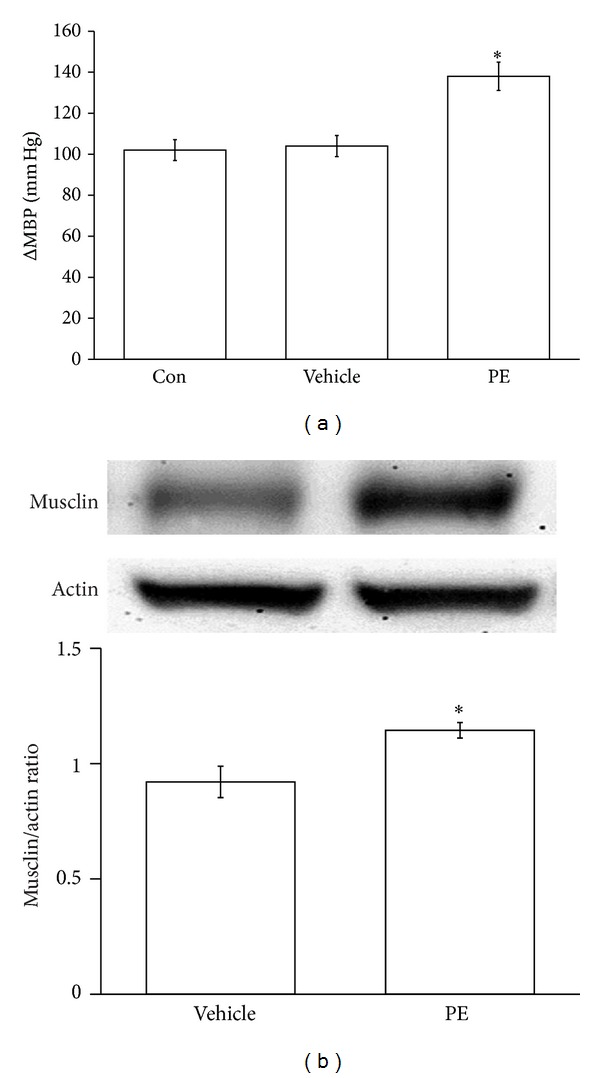
Effect of challenge with vasoconstrictor on the mean blood pressure and the expression of musclin protein. Wistar rats were injected intravenously (IV) with a vasoconstrictor named phenylephrine (PE; 10 *μ*g/kg) dissolved in 9% saline, the used vehicle, 4 times daily for one week. The mean blood pressure (MBP) was recorded using a noninvasive tail-cuff monitor (a), while the expression of musclin protein (11 kDa) was determined using Western blotting analysis (b). The quantification of the results is indicated as the means with the SE (*n* = 8 per group) in each column shown in the lower panel. *P < 0.05 compared to vehicle-treated group.

We examined the expression of musclin in the rat aorta of normotensive rats after repeated treatment with phenylephrine (PE) (four times a day) for one week. The mean blood pressure (MBP) after treatment with PE was significantly higher (Figure 2(a)) than the vehicle-treated group. PE also increased the expression of musclin protein in aorta isolated from treated animals (Figure 2(b)).

### 3.3. Effect of Phenylephrine on Musclin Expression in Rat Cell Line of Vascular Smooth Muscle Cells (A7r5 Cells)

**Figure 3:**
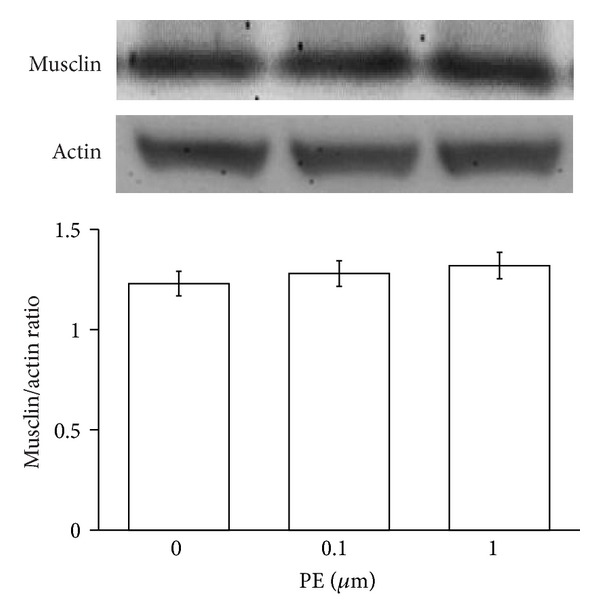
Effects of phenylephrine (PE) on the expression of musclin in A7r5 cells. Cells treated with PE for 24 hours were then harvested to measure the protein level of musclin expression using Western blotting analysis. All values are presented as mean ± SEM (*n* = 8). No difference was observed between all groups.

To identify the direct effect of PE on the expression of musclin, A7r5 cells were treated with PE. There is no difference in the expression of musclin in PE treated A7r5 cells (Figure 3).

### 3.4. Effect of Musclin on Arterial Strips Isolated from Rats

**Figure 4:**
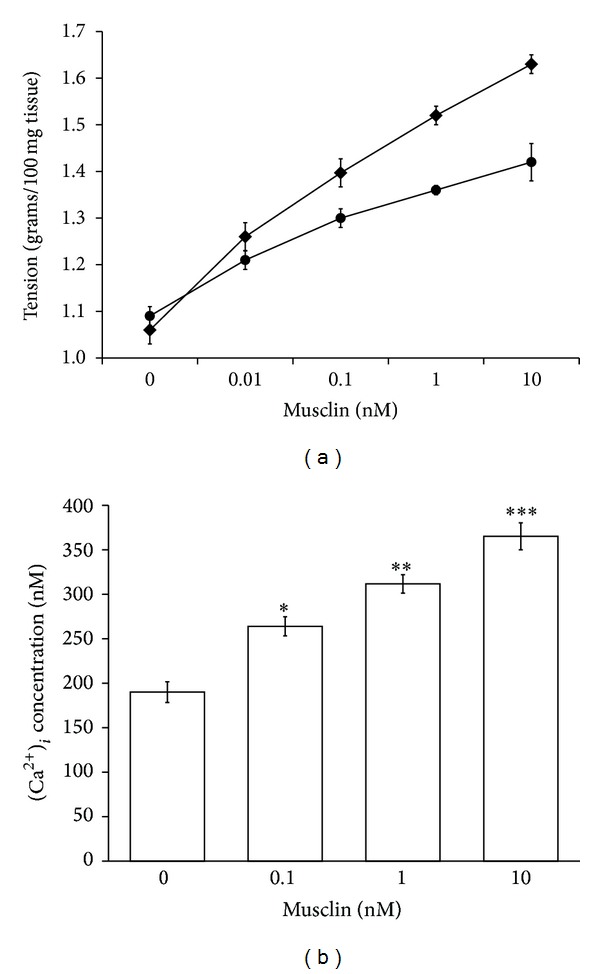
Effects of musclin on contraction of arterial strip and intracellular calcium in A7r5 cells. Vasoconstriction was induced in a concentration-dependent manner by musclin (0.01–10 nmol/L) in the arterial strips isolated from normal Wistar rats. The closed square showed the results in calcium-rich buffer while the closed circle showed the results in calcium-free buffer (a). Changes in intracellular calcium were detected with fura-2 by using a fluorescence spectrofluorometer. The cells were placed in buffered physiological saline solution with 5 *μ*M of fura-2-AM and incubated for 1 h. After recording the baseline value, musclin was added into the cuvette to detect the free intracellular calcium (b). All values are presented as mean ± SEM (*n* = 8). *P < 0.05, **P < 0.01, and ***P < 0.001 compared with the control group (musclin 0 nM).

Vasoconstriction was induced in a dose-dependent manner by musclin (0.01–10 nmol/L) in the arterial strips isolated from normal rats. However, the response to musclin in arterial strips was markedly reduced in calcium-free buffer as compared with that in calcium-rich buffer (Figure 4(a)). The action of musclin disappeared by washing the strips with normal buffer, and the response could be reproduced by the retreatment with musclin.

### 3.5. Changes of Intracellular Calcium Influx by Musclin in A7r5 Cells

The fluorescent probe, fura2-AM, was used to detect the changes in intracellular calcium level in A7r5 cells. Musclin (0.01–10 nmol/L) showed the significant increase of intracellular calcium level in a concentration-dependent manner (Figure 4(b)).

## 4. Discussion

In the present study, we found that expression of musclin is raised in arterial tissues isolated from DOCA-salt induced hypertensive rats. Also, similar increase of arterial musclin expression was observed in normal rats after repeated vasoconstriction challenge using phenylephrine. However, phenylephrine treated vascular smooth muscle cells (A7r5 cells) did not modify the expression of musclin. The direct effect of phenylephrine can thus be ruled out, and changes in the expression of musclin seem related mainly to the vasoconstriction. Furthermore, musclin induced a sustained vasoconstriction in the arterial strips isolated from normal rats in a concentration-dependent manner. The response was reduced when arterial strips were immersed in calcium-free buffer. Sustained increase of calcium influx by musclin was also characterized in A7r5 cells. Thus, to the best of our knowledge, this is the first study to show the higher expression of musclin in response to vasoconstriction that generally occurred in hypertension.

The administration of a synthetic mineralocorticoid derivative, DOCA, in combination with salt loading in the diet to young adult Wistar rats following surgical removal of one kidney induces hypertension with characteristic of human volume-overload induced hypertension [17–19]. DOCA-salt rats mimic most of the changes in human hypertension and vascular dysfunction [20]. In this study, we observed the higher expression of musclin in the arterial tissue of DOCA-salt induced hypertensive rats using Western blots. Thus, a higher expression of musclin is not only in the genetic animal model of SHRs but also in DOCA-salt induced hypertensive rats.

The phenylephrine (PE) induced vasoconstriction is widely used as an experimental model of hypertension [21, 22]. In this study, we found a higher expression of musclin in arterial tissue isolated from the PE treated rats using Western blots. However, an activation of *α*_1_-adrenoceptors by PE may influence the expression of genes and structure proteins [23]. Thus, we treated A7r5 cells with PE to investigate the direct effect of PE on the expression of musclin. However, PE did not modify the expression of musclin in cultured vascular cells. The higher expression of musclin in aorta seems related to vasoconstriction that generally occurred in hypertension.

Thus, an increase of aortic tone by hypertension may enhance the expression of musclin. Then, the raised expression of musclin could lead the hypertension to be more serious. For understanding the direct effect of musclin on aortic tone, the spirally cut aortic strips from normotensive rats were used. We gave up the use of aortic strips from SHRs that showed the pathologic state of vascular tone. Musclin induced sustained vasoconstriction was markedly reduced when arterial strips were immersed in calcium-free buffer. It has been indicated that vasoconstrictors may cause intracellular calcium release from intracellular calcium pool to develop the tension of arterial smooth muscle [24, 25]. Thus, the vasoconstriction induced by musclin seems not dependent on calcium influx only. Release of calcium from intracellular pool is also involved in the vasoconstriction of musclin.

Calcium ions are essential for muscle contraction while calcium influx is the major pathway to increase intracellular calcium. [26–29]. Modulation of myofilament properties by alterations in the calcium concentration has profound effects on smooth muscle contractility [28]. In the present study, we demonstrated the vasoconstriction of musclin through calcium influx and an increase of intracellular calcium by musclin in A7r5 cells. Thus, musclin may increase vascular tone through enhancement of intracellular calcium that is important in the development of arterial hypertension.

The vasoconstriction induced by musclin appeared to be calcium dependent. However, intracellular signals for the action of musclin are still unclear. It has been suggested that musclin may activate its specific receptors and NPR-C receptors in cardiovascular tissues and cells [9]. Also, it has been established that ANP interacts with NPR-C in aorta to activate calcium-loaded calmodulin and others for vasoconstriction [30]. Thus, the potential mechanisms of musclin induced vasoconstriction could be elucidated in a calcium-dependent manner. But the real signals need more investigations in the future.

## 5. Conclusion

In the present study, we observed a higher expression of musclin in aorta during hypertension. Also, musclin contributed to the development of hypertension through increase of intracellular calcium in vascular smooth muscle. Thus, we suggest that musclin could be considered as a new target in the development of agent(s) for treatment of hypertension.
